# Anti-Tumoral Effect and Action Mechanism of Exosomes Derived From *Toxoplasma gondii*-Infected Dendritic Cells in Mice Colorectal Cancer

**DOI:** 10.3389/fonc.2022.870528

**Published:** 2022-04-22

**Authors:** Shilan Zhu, Jinmiao Lu, Zhibing Lin, Asmaa M. I. Abuzeid, Xiaoyu Chen, Tingting Zhuang, Haiyan Gong, Rongsheng Mi, Yan Huang, Zhaoguo Chen, Guoqing Li

**Affiliations:** ^1^Guangdong Provincial Key Laboratory of Zoonosis Prevention and Control, College of Veterinary Medicine, South China Agricultural University, Guangzhou, China; ^2^Key Laboratory of Animal Parasitology of Ministry of Agriculture, Laboratory of Quality and Safety Risk Assessment for Animal Products on Biohazards (Shanghai) of Ministry of Agriculture, Shanghai Veterinary Research Institute, Chinese Academy of Agricultural Sciences, Shanghai, China; ^3^School of Agriculture and Biology, Shanghai Jiao Tong University, Shanghai, China; ^4^Faculty of Veterinary Medicine, Suez Canal University, Ismailia, Egypt

**Keywords:** *Toxoplasma gondii*, dendritic cells, exosome, miRNA, macrophage, miR-155-5p

## Abstract

*Toxoplasma gondii* is an obligate intracellular protozoan with anti-tumor activity against a variety of cancers. However, the therapeutic effect of *T. gondii* on colorectal cancer is unclear, and using direct *Toxoplasma* infection in immunotherapy involves safety concerns. This study investigated the anti-tumoral effect and mechanism of exosomes derived from dendritic cells (DCs) infected with *T. gondii* (Me49-DC-Exo). We used differential ultracentrifugation to isolate exosomes from uninfected DCs (DC-Exo) and *T. gondii* Me49-infected DCs (Me49-DC-Exo). The isolated exosomes were identified by transmission electron microscopy, nanoparticle tracking analysis, and western blotting. Me49-DC-Exo significantly inhibited the tumor growth and reduced the proportion of M2 macrophages in the blood of tumor-bearing mice. *In vitro*, Me49-DC-Exo suppressed macrophage (RAW264.7) polarization to M2 phenotype. miRNA sequencing revealed that multiple miRNAs in Me49-DC-Exo were differentially expressed compared with DC-Exo, among which miR-182-5p, miR-155-5p, miR-125b-2-3p, and miR-155-3p were up-regulated, while miR-9-5p was significantly down-regulated. Transfecting mimics or inhibitors of these differential miRNAs into RAW264.7 cells showed that miR-155-5p promoted M1 macrophage polarization while inhibiting M2 macrophage polarization. Bioinformatics prediction and dual-luciferase reporter assay confirmed the suppressor of cytokine signaling 1 (SOCS1) as a direct target of miR-155-5p. Silencing SOCS1 gene expression in RAW264.7 cells increased CD86 ^+^ CD206 ^−^ M1 macrophage proportion, and inducible nitric oxide synthase and tumor necrosis factor-α mRNA levels. However, arginase-1 and transglutaminase 2 expression levels decreased. These results suggest that the exosomes inhibit macrophage polarization to M2 phenotype and regulate SOCS1 expression by delivering functional miR-155-5p. These findings provide new ideas for colorectal cancer immunotherapy.

## Introduction

Colorectal cancer (CRC) is one of the most common cancers and is the third-largest cause of cancer death globally (1). The induction of anti-tumor immunity has proven to be an effective strategy for cancer therapy (2). Tumor-associated macrophages (TAMs) are the most abundant immunosuppressive cells involved in tumors. These cells are activated by microenvironment signals to produce different functional phenotypes (3). TAMs are generally divided into two phenotypes: M1 (classical activation) and M2 (alternative activation) macrophages (4). M1 macrophages are usually activated by Toll-like receptor-4 (TLR4), lipopolysaccharide (LPS), granulocyte-macrophage colony-stimulating factor (GM-CSF), or Th1 cytokines, such as interferon-gamma (IFN-*γ*) (5). Additionally, M1 macrophages are typically characterized by high expression of major histocompatibility complex (MHC) class II receptor and co-stimulatory molecules, such as CD86, and the production of pro-inflammatory cytokines (Interleukins IL-1β, IL-12, IL-23, and tumor necrosis factor (TNF-*α*), reactive oxygen species, and nitrogen intermediates (6). Therefore, M1 macrophages play an essential role in promoting inflammatory response and anti-tumor immunity (7). M2 macrophages are activated by Th2 cytokines, such as IL-4, IL-10, and IL-13 (8), and have unique surface molecule CD163 and CD206 (9). These macrophages also express high levels of arginase1 (Arg-1) and inhibitory cytokines (IL-10 and TGM-*β*), which are key cellular components of anti-inflammatory and pro-tumor activity. The phenotype of each macrophage is not fixed and can be dynamically shifted between M1 and M2 phenotype (10, 11). TAMs usually exhibit M2 phenotype and promote tumor progression by mediating immunosuppression and angiogenesis.

Exosomes are lipid-bilayered vesicles with a 40–200 nm diameter secreted by cells (12) and contain various bioactive substances, such as proteins, lipids, DNA fragments, and microRNAs (miRNAs). The composition of exosomes is highly regulated by cellular origin and vary according to different physiological or pathological conditions (13). The exosome contents can be released into recipient cells to mediate intercellular communication (14). Recently, the role of exosomes in regulating macrophage polarization to promote or inhibit the growth and metastasis of tumor has been frequently reported (15, 16). Glioblastoma stem cell-derived exosomes are enriched in phosphorylated signal transducer and activator of transcription 3 (STAT3) and skewed macrophages toward the M2 phenotype (17). Blood-derived exosomes from prostate cancer patients induced macrophage polarization to tumor-promoting M2 phenotype *via* milk fat globule-EGF factor 8 (MFG-E8)-mediated efferocytosis (18). Long non-coding RNA (LncRNA) TUC339 carried by exosomes derived from hepatoma cells played a key role in regulating the polarization of macrophages toward the M2 phenotype (19). Moreover, macrophage polarization can be controlled by exosomes that contain miRNA as a crucial component (13). miRNAs are a class of 19-23 nucleotides, single-stranded non-coding RNAs that bind to the 3’UTR region of target gene mRNA and regulate gene expression at the post-transcriptional level, playing a crucial role in regulating most biological processes. Endometrial cancer cells-derived exosomal miR-21 induced macrophages to polarize into M2 phenotype under hypoxia conditions (20). In pancreatic ductal adenocarcinoma, tumor-derived exosomes could affect macrophage polarization through the microRNA-501-3p/TGFBR3 signaling pathway (21).

Parasitic infections have demonstrated inhibitory effects on tumor growth. *Toxoplasma gondii* is an obligate intracellular parasite that stimulates anti-tumor immune activity in multiple cancer types. In mouse model of Lewis lung cancer, *T. gondii* infection inhibited tumor growth by inducing Th1 immune response and antiangiogenic activity (22). Formalin-fixed *T. gondii* induced potent anti-tumor activities in Lewis lung cancer and EL4 lymphoma (23, 24). In a mouse model of xenograft breast cancer, injection of live *T. gondii* suppressed tumor progression (25). However, the safety of immunotherapy with direct infection of live *T. gondii* is questionable. Thus, an alternate strategy for tumor therapy with *T. gondii* immune modulation is urgently needed.

Here, we tested the therapeutic effect of exosomes (Me49-DC-Exo) isolated from dendritic cells (DC) infected with *T. gondii* Me49 strain in a mice model of CRC. The potential mechanism was explored by sequencing miRNA in exosomes, bioinformatics prediction of target genes, dual-luciferase experimental verification, and RNA interference assay. This study would provide new ideas for the immunotherapy of colorectal cancer.

## Materials and Methods

### Ethical Approval

All animal experiments were approved by the Animal Care and Use Committee of Shanghai Veterinary Research Institute, the Chinese Academy of Agricultural Sciences (ethical approval number: SHVRI-SZ-20200923-01)

### Parasites, Cells, and Animals

*Toxoplasma gondii* Me49 strain was preserved in our laboratory and maintained by serial passage in human foreskin fibroblasts (HFF). RAW264.7 cell line (laboratory preservation) was cultured in Dulbecco^’^s Modified Eagle’s Medium (DMEM), supplemented with 10% fetal bovine serum (FBS) and 1% penicillin-streptomycin (PS). A mouse colon cancer cell line stably expressing the firefly luciferase reporter gene (CT26-CMV-Luc-Puro) was purchased from Heyuan Biotechnology (Shanghai) Co., Ltd. and cultured in RPMI-1640 medium supplemented with 10% FBS and 1% PS at 37°C and 5% CO_2._ Specific pathogen-free (SPF) female BALB/c mice aged 6-8 weeks were purchased from Sibafu (Beijing) Biotechnology Co., Ltd.

### DC Isolation and Culture

Bone marrow cells were isolated from the tibia and femur of wild-type C57/BL6J mice aged 4-5 weeks. After euthanasia of mice, a 1 mL syringe was used to inject sterile PBS to repeatedly wash the bone marrow cavity to collect bone marrow cells. The isolated cells were filtered through a 70 μm filter to obtain a single cell suspension and mixed with 8 mL 1 × Red Blood Cell lysis buffer and let to stand for 5 min to lyse RBCs. The cell suspension was washed twice with phosphate buffer saline (PBS) and centrifuged at 1000 rpm for 5 min at room temperature. Cells were counted and seeded at a density of 1 × 10^7^ cells in a 10 cm cell culture plate. Then, a 10 mL of RPMI 1640 complete medium was added to each plate and incubated at 37°C, 5% CO_2_ in a cell incubator. This RPMI 1640 medium contained 10% exosome-depleted FBS (prepared by ultracentrifugation at 100,000 g for 12 h), 1% PS, 10 ng/mL granulocyte-macrophage colony-stimulating factor (GM-CSF) and 10 ng/mL interleukin 4. RPMI 1640, FBS, and PS were purchased from Gibco, ThermoFisher Scientific (Waltham, MA, USA), while GM-CSF and IL-4 were obtained from PeproTech Inc. (Cranbury, NJ, USA). After that, fresh medium was replaced every two days until cells differentiated into dendritic cells at 8-9 days.

### Isolation of Exosomes

*T. gondii* Me49 strain was incubated with DC/RPMI 1640 culture containing 10% exosome-depleted FBS and 1% PS for 12 h at 37°C, 5% CO_2_. *T. gondii* Me49-infected DC and uninfected DC culture supernatants were collected by centrifugation at 500 g for 10 min. The supernatants were centrifuged at 16,500 g for 30 min, filtered through a 0.22 μm filter, and then centrifuged at 120,000 g for 90 min to precipitate exosomes. The pellets were resuspended in 200 μL of PBS after being washed with PBS and centrifuged at 120,000 g for 90 min twice. All the above-mentioned centrifugal steps were carried out at 4°C.

### Identification of Exosomes

The isolated exosomes were characterized using transmission electron microscopy (TEM), nanoparticle tracking analysis (NTA), and western blotting (WB). The TEM procedures were as follows: 8 μL of enriched exosomes sample was dropped onto a 200-micron copper mesh and let to stand for 45 s at room temperature. Following draining excess liquid from the edge of copper mesh with dry filter paper, 8 μL of 3% phosphotungstic acid was added for negative staining for 45 s. After air drying at room temperature, the morphology of exosomes was observed at 80 kV under the Tecnai G2 Spirit Bio Twin Transmission Electron Microscope (FEI Company, Hillsboro, OR, USA). The exosome particle size distribution was analyzed using a ZetaView Nanoparticle Tracking Analyzer (Particle Metrix, Meerbusch, Germany). After calibrating with the standard solution, the sample pool was rinsed with PBS to remove residual particles, and the exosomes obtained by ultracentrifugation were diluted 100 times with PBS and injected into the measurement sample pool. Five different fields were selected for measurement, and the final particle size output data were combined and averaged to generate the final particle size distribution and concentration for each sample. The expressions of CD9, CD63, and tumor susceptibility gene 101 (TSG101) in isolated exosomes were examined by WB. Briefly, 6 × Sodium Dodecyl Sulfate (SDS) loading buffer was added to exosome samples, boiled at 100°C for 10 min, and then separated by SDS-PAGE (Polyacrylamide Gel-Electrophoresis). The proteins were electro-transferred from gels into poly vinylidene fluoride (PVDF) membranes in a tris-glycine-methanol buffer. Then, the membrane was blocked with 5% skimmed milk in 1 × PBST (phosphate buffer saline-Tween) solution for 1 h. PVDF membranes containing protein were incubated overnight at 4°C with rabbit anti-mouse CD9, CD63 (1:2000, Abcam, Cambridge, MA, USA), and mouse anti-TSG101 (1:2000, Abcam) as primary antibodies. The used corresponding secondary antibodies were horseradish peroxidase (HRP)-labeled goat anti-rabbit IgG antibody (1:2000, Abcam) and goat anti-mouse IgG antibody (1:2000, Abcam). The electrochemiluminescence (ECL) method was used to detect WB reactions.

### Treatment Model of Colon Cancer-Bearing Mice

After adapting wild-type BALB/c mice aged 6-8 weeks in an SPF animal chamber for one week, 5×10^5^ CT26 cells (mouse colon cancer cell line) were subcutaneously injected into the right side of each mouse to establish a mouse model of xenograft colon cancer. At 48 h after injection, the mice were randomly divided into three groups (five mice/group). The three mice groups were injected intratumorally with PBS, DC-Exo, and Me49-DC-Exo (5 mg/mice), respectively. The same treatment was repeated twice every other day. Tumor growth was monitored using *in vivo* imaging techniques, and bioluminescent signals were quantified using the IVIS^®^ Spectrum *In Vivo* Imaging System (PerkinElmer Inc., Waltham, MA, USA).

### Evaluation of M1/M2 Macrophage Expression by Flow Cytometry

After exosome treatment, mouse whole blood samples were collected from the eyeball in anticoagulant tubes. A 8 mL of erythrocyte lysis buffer was added and mixed to lyse RBCs for 10 min at room temperature. Then, the cell precipitate was collected by centrifugation (600 g, 4°C, 5 min) and washed twice with 10 mL PBS for centrifugating (600 g, 4°C, 10 min) and discarding supernatants. Later, the cell precipitate was resuspended in a blocking solution (1: 200 ratio of healthy mouse serum and DPBS, Dulbecco’s phosphate-buffered saline, 500 µL/tube) and incubated at 4°C for 30 min. Antibody labeling was performed after blocking. Cells of each group were divided into six Eppendorf tubes (five tubes for staining and one blank tube). After blocking, each tube of cells was washed with 1 mL staining buffer and centrifuged (600g, 4°C, 5 min) to discard supernatant, and then cells were resuspended in 200 µL staining buffer (FBS and DPBS with 1:100 ratio). Tubes for CD206 staining were left aside for a while. Cell surface markers were stained by incubating resuspended cells with fluorescent antibodies at 4°C for 30 min in the dark. The used antibodies were fluorescein isothiocyanate (FITC) anti-mouse CD45, phycoerythrin (PE) anti-mouse CD11b, pacific blue450-brilliant violet421 (PB450-BV421) anti-mouse F4/80, and PE-Cy (Cyanine)7 anti-mouse CD86 antibodies (1:100, BD Biosciences, Franklin Lakes, NJ, USA). After cell surface marker staining, cells were washed twice with staining buffer (200 µL) and centrifuged at 600 g for 5 min. The cells used for intracellular staining (CD206 staining) were permeabilized with BD Cytofix/Cytoperm™ fixation/permeabilization solution (BD Biosciences, 250 µL/tube) at 4°C for 20 min. After washing twice with 1 × BD Perm/Wash^™^ buffer (BD Biosciences, 1 mL/tube) and centrifugation (600 g, 5 min), cells were incubated with 5 µL of allophycocyanin (APC) anti-mouse CD206 antibodies (1:50, BD Biosciences) diluted in 550 µL of BD Perm/Wash^™^ buffer at 4°C for 30 min for intracellular antigen staining. After centrifugation, the cell precipitate was washed twice with 1× BD Perm/Wash^™^ buffer and centrifuged at 600 g for 5 min. Finally, the cells were analyzed by the Cytoflex instrument (Beckman Coulter, Hialeah, FL, USA).

### Labeling and Tracing of Exosomes

DC-Exo and Me49-DC-Exo were labeled with Mem Dye-Green green fluorescent membrane dye (EX01 ExoSparkler Exosome Membrane Labeling Kit-Green, Dojindo, Japan) according to the kit instructions. The labeled exosomes were added to RAW264.7 cells and incubated for 18 h. The cells were collected and washed twice with PBS. The nuclei of RAW264.7 cells were stained with Hoechst dye (Sigma-Aldrich, Taufkirchen, Germany) for 15 min at room temperature. After washing three times with PBST, the slides were mounted with an anti-fluorescence quenching mounting medium and observed using Zeiss LSM880 Confocal Laser Scanning Microscope (Zeiss, Jena, Germany).

### High-Throughput Sequencing Analysis

Small RNAs in exosomes were sequenced by Shanghai Paiseno Biotechnology Company. The procedures were as follows: three groups of DC-Exo and Me49-DC-Exo were randomly selected. Total RNA was extracted from samples using exoRNeasy Serum/Plasma Maxi Kit (Qiagen, Hilden, Germany). The purity and integrity of total RNA were detected by an Agilent 2100 Bioanalyzer (Agilent, Santa Clara, CA, USA). TruSeq Small RNA Sample Prep Kit was used to construct small RNA libraries from RNA samples that passed quality control. After enrichment, purification, quality inspection, and quantification, six sets of samples were sequenced using the Illumina HiSeqTM 2500 sequencing platform (Illumina, San Diego, CA, USA). Raw data have been deposited at the Gene Expression Omnibus (GEO) (http://www.ncbi.nlm.nih.gov/geo/) under the accession number GSE 197114.

### Quantitative Reverse-Transcription Polymerase Chain Reaction (qRT-PCR)

Total RNA was extracted from cells or exosomes using Trizol reagent. First-strand cDNA was synthesized from total RNA samples meeting purity and integrity requirements using the PrimeScript RT reagent Kit (TaKaRa, Yishan Huitong Technology Co., Ltd, Beijing, China). The qRT-PCR was performed to evaluate miRNA expression level in exosomes as well as inducible nitric oxide synthase (INOS), tumor necrosis factor-alpha (TNF-α), interferon regulatory factor 5 (IRF5), arginase-1 (Arg-1), interleukin 10 (IL-10), transglutaminase 2 (TGM2), and suppressor of cytokine signaling 1 (SOCS1) genes in cells. U6 snRNA and GAPDH were used as the corresponding internal reference genes. All primer sequences used for qPCR are listed in **Table S1 of Appendix 1**. The reaction systems (total 20 μL) contained 2 μL of cDNA, 0.4 μL of each forward and reverse primers, 0.4 μL of passive reference dye, 10 μL of 2 × SYBR Green mix (TaKaRa, Japan), and 6.8 μL of double-distilled water. Reaction conditions were pre-denaturation at 94°C for 30 s and 40 cycles of 94°C for 5 s and 60°C for 30 s. Relative expression levels of miRNA and target genes were calculated by the 2^-ΔΔCT^ method (26).

### Transient Transfection

RAW264.7 cells/DMEM were seeded in a 6-well culture plate at a density of 4 × 10^5^cells/well (80% confluent). A 2 μL Lipofectamine^®^ 2000 transfection reagent (Gibco, ThermoFisher Scientific, Waltham, MA, USA) was diluted in a 50 μL of Opti-MEM^®^ medium (Gibco) and incubated for 5 min at room temperature. A 2 μL of miR-155-5p mimics (20 μM, Genepharma, Shanghai, China), miR-155-5p inhibitors (20 μM, Genepharma) or miRNA negative control (NC) were diluted in 50 μL of Opti-MEM^®^ medium/each. Diluted miR-155-5p inhibitors/mimics/NC were gently mixed with diluted Lipofectamine^®^ 2000 Reagent (1:1 ratio) and incubated for 20 min at room temperature. Simultaneously, the seeded RAW264.7 cells were washed with DMEM free from serum and antibiotics twice, and then 400 μL serum/antibiotic-free DMEM was added per well. Then, the incubated miRNA-transfection reagent complex (100 μL/well) was mixed with the RAW264.7 cells in the plate. After incubation at 37°C, 5% CO_2_ for 6 h, serum/antibiotic-free DMEM was replaced with complete DMEM, and the cultivation continued for 48 h. Cells in each group were collected for subsequent experiments. SOCS1 small interfering RNA (siRNA) was synthesized by Genepharma company (Shanghai, China). The sequences of SOCS1- siRNA and siRNA negative control were 5′-ACACTCACTTCCGCACCTT-3′ and 5′-CAGCCTTCCTTCTTGGGTAT-3′, respectively. RAW264.7 cells (80% confluent) were inoculated with the incubated mixture of 2 μL Lipofectamine 2000 transfection reagent (diluted in 50 μL Opti-MEM^®^ medium) and 5 μL siRNA (diluted in Opti-MEM^®^ medium, final concentration 25 nM). After culturing for 6 h in serum/antibiotic-free DMEM, the medium was replaced with DMEM complete medium and continued incubation for 48 h. Then, cells were collected for subsequent experiments. We used flow cytometry and qRT-PCR to analyze the M1/M2 macrophage-related markers and gene expression in transfected RAW264.7 cells from all groups using the methods mentioned above.

### Prediction of Target Genes and Double Luciferase Reporter Assay

The target genes of miR-155-5p were predicted by the online software Targetscan (http://www.targetscan.org/). The 3’UTR fragment of the target gene (SOCS1) containing miR-155-5p complementary sequence was amplified by PCR. Primers used in PCR amplification of the wild-type fragment of SOCS1 3’UTR region are F: 5’- TGTTTAAACGAGCTCGCTAGCCAGCGCCGCGTGCGGCCG-3’ and R: 5’-CAGGTCGACTCTAGACTCGAGCTACAACCAGGGGGGACCC-3’. Primers used in PCR amplification of two fragments of the mutated SOCS1 3’UTR region are F1: 5’- TGTTTAAACGAGCTCGCTAGCCAGCGCCGCGTGCGGCCG -3’ and R1: 5’-AAATAATAAGGCGCCCCCACTTCCTCAT-3’, and F2: 5’-CATGAGGAAGTGGGGGCGCCTTATTAT-3’ and R2: 5’- CAGGTCGACTCTAGACTCGAGCTACAACCAGGGGGGACCC-3’, respectively. PCR reaction consisted of 2 × EmeraldAmp PCR Master Mix (25 µL, TaKaRa, Dalian, China), forward primer (1 µL), reverse primer (1 µL), template DNA (100 ng), and ddH_2_O (up to 50 µL). PCR cycling program was as follows: 94°C for 3 min; 34 cycles of 94°C for 30 s, 58°C for 30 s, and 72°C for 24 s (12 s for amplification of partial fragment); and 72°C for 5 min. PCR products were examined on 1% agarose gel electrophoresis and then purified using the E.Z.N.A.^®^ Gel Extraction Kit (Omega Bio-Tek, Norcross, GA, USA). Purified PCR product and the plasmid vector pmirGLO^®^ (Promega, Madison, WI, USA) were double-digested with *Nhe*I and *Xba*I and ligated to construct the wild-type dual-luciferase reporter gene vector (WT-SOCS1-3’UTR). A mutant dual-luciferase reporter gene vector (MUT-SOCS1-3’UTR) was constructed by mutating the seed region of the miR-155-5p binding site. Then, 2 μg of dual-luciferase reporter gene vector and 100 nM miR-155-5p mimic or miRNA negative control were co-transfected into human embryonic kidney (HEK)-293T cells. At 48 h after transfection, the cells were collected and washed three times with PBS and analyzed using a dual-luciferase reporter assay system (Promega, Madison, WI, USA) following the manufacturer^’^s protocol. A 500 μL of cells lyzed by Passive Lysis Buffer was added to each well of a six-well culture plate. The luciferase activity was analyzed after shaking for 15 min. Firefly luciferase activity was normalized by Renilla luciferase activity.

### Statistical Analysis

SPSS 21.0 (IBM Corp, Armonk, NY, USA) was used for statistical analysis of data, which were expressed as mean ± standard deviation. The independent sample t-test was used to analyze the differences between two groups with normal distribution and equal variances. When the variance did not have a normal distribution, the nonparametric Mann-Whitney U test was used. Differences between groups were analyzed using the one-way analysis of variance (ANOVA) followed by Tukey’s multiple comparison test. Statistical difference was considered when *p* < 0.05.

## Results

### Isolation and Identification of Exosomes

**Figure 1:**
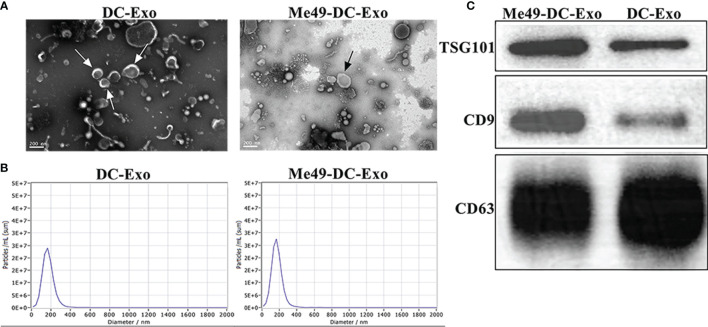
Isolation and identification of exosomes. **(A)** Exosomes observed by TEM (80 KV, 30 K). **(B)** Size and distribution of exosomes analyzed by NTA. **(C)** Specific markers of exosomes identified by WB.

Transmission electron microscopy was used to examine the morphology of Me49-DC-Exo/DC-Exo. Both exosomes showed continuous bilayer membranes with a saucer-like structure (**Figure 1A**). Particle size analysis showed that the particle size of exosomes was mainly distributed at 100-200 nm (**Figure 1B**), which is compatible with the general morphological feature and the size of exosomes. Western blot detected the expression of exosomal-specific markers, CD9, CD63, and TSG101(**Figure 1C**).

### Me49-DC-Exo Inhibited Tumor Growth and Regulated Macrophage Polarization

**Figure 2:**
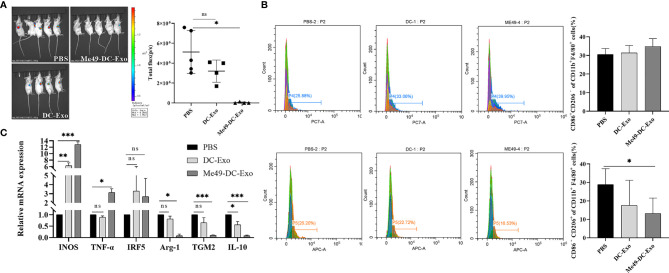
Me49-DC-Exo inhibited tumor growth in mouse and regulated macrophage polarization. **(A)** On day 4 after treatment, the IVIS imager detected bioluminescence images in tumors of mouse and quantified the bioluminescence signal intensity of each tumor in mouse. **(B)** Flow cytometry was used to label CD86 ^+^ or CD206 ^+^, and CD45 ^+^ CD11b ^+^ F4/80 ^+^ macrophages in blood of mice injected with DC-Exo and Me49-DC-Exo were stained to detect the percentage of CD86^+^ CD206 ^−^ M1 macrophages and CD86 ^−^ CD206 ^+^ M2 macrophages. **(C)** mRNA levels of M1 macrophage specific genes (INOS, TNF-α, and IRF5) and M2 macrophage specific genes (TGM2, Arg-1 and IL10) in blood of tumor-bearing mice injected intratumorally with PBS, DC-Exo and Me49-DC-Exo were detected by qRT-PCR. The data were expressed as mean ± standard deviation, and the independent sample t-test was used to compare the statistical differences between two groups. ns (*p* ≥ 0.05), *p < 0.05, **p < 0.01, ***p < 0.001.

A tumor -bearing mouse model was established to investigate the effect of exosomes on the growth of colon cancer (CT26-luc tumor). *In vivo* imaging analysis identified a strong fluorescent signal in the tumor sites of the tumor-bearing mice treated with PBS and DC-Exo. By contrast, no fluorescent signal was detected in the tumor sites of the tumor-bearing mice treated with Me49-DC-Exo, indicating that tumor growth was significantly inhibited (**Figure 2A**).

To investigate whether exosomes inhibit tumor growth by affecting the polarization of macrophages, tumor-bearing mice were treated with DC-Exo, PBS, and Me49-DC-Exo. The proportion of M1 and M2 macrophages in the blood of tumor-bearing mice of all treatments was analyzed by flow cytometry. M2 macrophages expressing CD86^−^ CD206^+^ in the Me49-DC-Exo group were significantly reduced compared with tumor-bearing mice treated with PBS (*p* < 0.05) (**Figure 2B**), suggesting that exosomes from *T. gondii*-infected DC could significantly inhibit macrophage polarization to M2 phenotype.

The mRNA levels of INOS, TNF-*α*, IRF5, IL-10, TGM2, and Arg-1 in the blood of tumor-bearing mice treated with the exosomes were detected by qRT-PCR. The mRNA levels of M1 macrophage-specific genes in the blood of tumor-bearing mice treated with DC-Exo and Me49-DC-Exo were higher than in the PBS group. Still, the statistical differences were more significant in the me49-DC-Exo group (**Figure 2C**). IL-10, TGM2, and Arg-1 expression levels in the Me49-DC-Exo group were significantly lower than those in the PBS group. The same genes were also downregulated in the DC-Exo group but without a significant difference (**Figure 2C**). These data indicate that Me49-DC-Exo promotes the expression of M1 macrophage-specific genes and inhibits the expression of M2 macrophage-specific genes. Meanwhile, these results suggest that Me49-DC-Exo may promote M1 polarization of macrophages and inhibit M2 polarization of macrophages.

### Uptake of DC-Exo and Me49-DC-Exo by Macrophages

**Figure 3:**
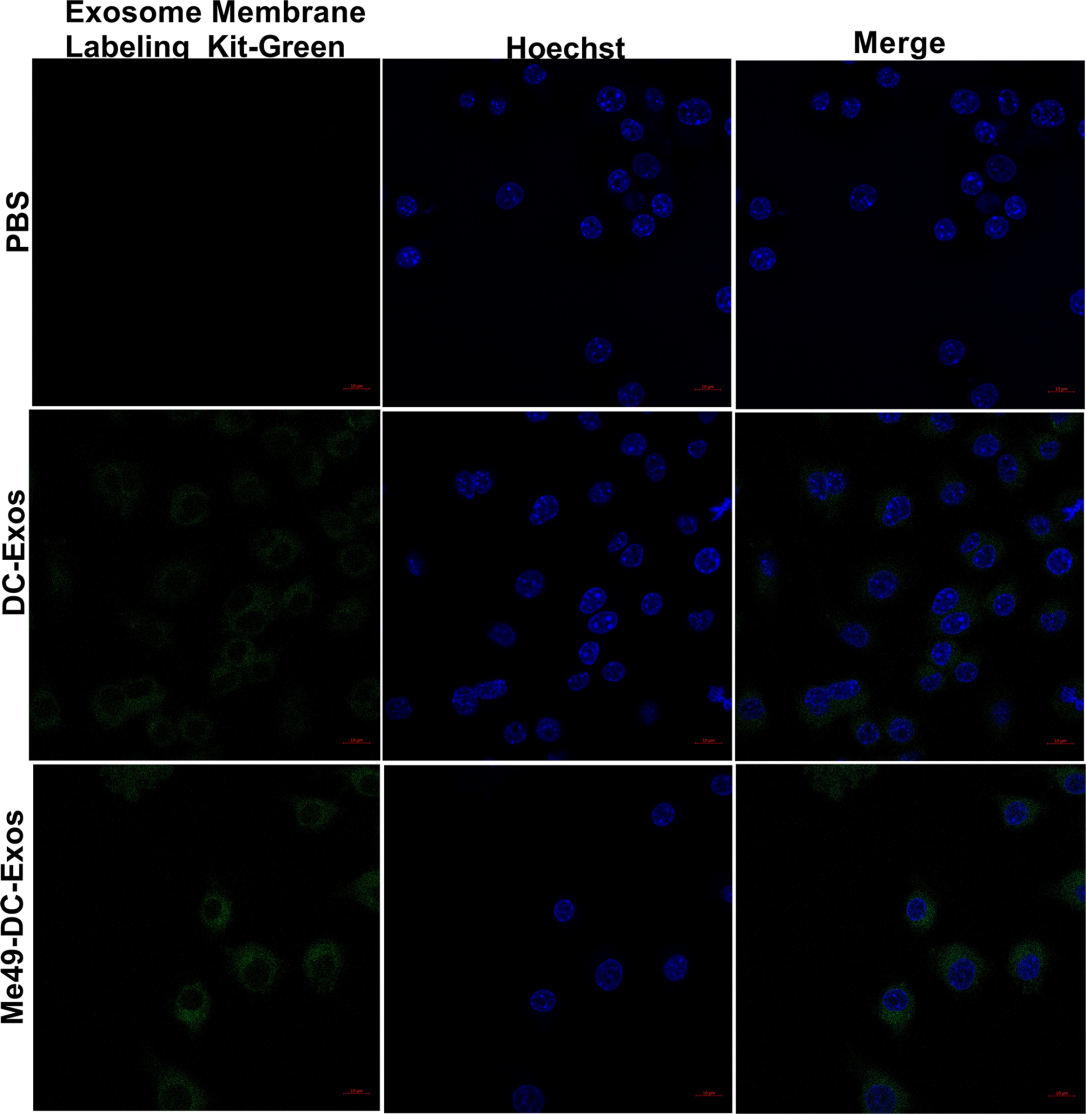
Verification of exosome uptake by macrophages. PBS control without exosomes, Mem Dye-Green labeled DC-Exo and Me49-DC-Exo were added to macrophage Raw264.7 and observed under laser confocal microscope. In the fluorescence microscope image, Mem Dye-green and Hoechst was used to label exosomes (Green) and macrophage nuclei (blue). Each experiment was repeated three times.

Exosomes can alter the function and activity of recipient cells. To confirm macrophage uptake of exosomes, we labeled exosomes and nuclei of macrophages (RAW264.7) with Mem Dye-Green dye (green) and Hoechst dye (blue), respectively. Punctured green fluorescence was noticed around the nuclei of macrophages treated with DC-Exo and Me49-DC-Exo (**Figure 3**), demonstrating that these exosomes could be successfully internalized by RAW264.7 cells.

### Me49-DC-Exo Promoted M1 Polarization of Macrophages *In Vitro*

**Figure 4:**
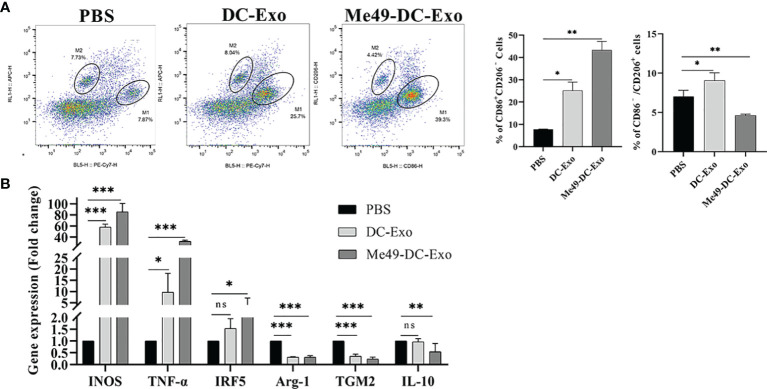
Regulation of macrophage polarization by Me49-DC-Exo. **(A)** CD86^+^ CD206^–^ M1 and CD86^–^CD206^+^ M2 macrophages were detected by flow cytometry, and the percentage of CD86 ^+^ CD206 ^−/^CD86 ^−^ CD206 ^+^ positive macrophages was quantified. **(B)** The expression levels of characteristic genes of M1 and M2 macrophage in Raw264.7 cells treated with exosome were analyzed by qRT-PCR. ns (*p* ≥ 0.05), *p < 0.05, **p < 0.01, ***p < 0.001.

To identify how Me49-DC-Exo regulates macrophage polarization, RAW264.7 cells were treated by DC-Exo, Me49-DC-Exo, or PBS for 24 h. Then, the proportion of M1 and M2 macrophages was analyzed by flow cytometry. The results showed that the proportion of M1 macrophages in the Me49-DC-Exo group was significantly increased (*p* < 0.01), while the proportion of M2 macrophages was significantly decreased (*p* < 0.01) (**Figure 4A**). qRT-PCR was used to evaluate M1 and M2 macrophage-specific gene expression levels. Me49-DC-Exo significantly inhibited the expression of M2 characteristic genes (TGM2 and IL-10) (*p* < 0.05) but promoted the expression of M1-specific genes (INOS and TNF-α) (*p* < 0.001) (**Figure 4B**). These data suggest that the exosomes could regulate the polarization of RAW264.7 cells to M1 macrophages *in vitro*.

### miRNA Profiles of DC-Exo and Me49-DC-Exo

**Figure 5:**
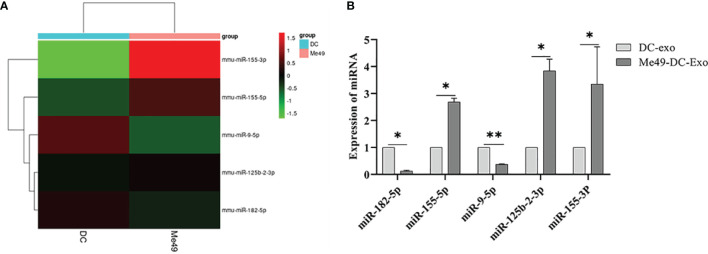
High-throughput sequencing of total RNA extracted from DC-Exo and Me49-DC-Exo. **(A)** Heatmap of miRNA differentially expressed in DC-Exo and Me49-DC- Exo. **(B)** The expression levels of differential miRNA verified by qRT-PCR. *p < 0.05, **p < 0.01.

We analyzed miRNA profiles by high-throughput sequencing to explore the role of exosomal miRNA in macrophage polarization. Compared to DC-Exo, a total of five miRNA with stable and significantly different expressions were detected in Me49-DC-Exo. The differentially expressed miRNAs were miR-182-5p, miR-155-5p, miR-9-5p, miR-125b-2-3p, and miR-155-3p (**Figure 5A**). qRT-PCR verified the expression levels of these five miRNAs. miR-182-5p, miR-155-5p, miR-125b-2-3p, and miR-155-3p were significantly up-regulated in the Me49-DC-Exo group compared with DC-Exo group (*p* < 0.05), while miR-9-5p was significantly down-regulated (*p* < 0.01) (**Figure 5B**).

### miR-155-5p Promoted M1 Polarization of Macrophages

**Figure 6:**
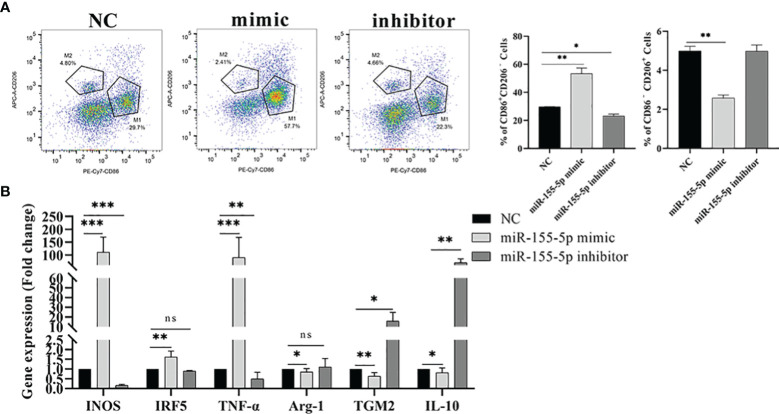
Regulation of macrophage polarization by miR-155-5p. **(A)** CD86 ^+^ CD206 ^−^ and CD86 ^−^ CD206 ^+^ positive macrophages were detected by flow cytometry, and the percentage of CD86 ^+^ CD206 ^−^ M1 and CD86 ^−^ CD206 ^+^ M2 macrophages was quantified. **(B)** The expression levels of characteristic genes of M1 and M2 macrophages in Raw264.7 cells treated with miR-155-5p mimics and inhibitors were analyzed by qRT-PCR. ns (*p* ≥ 0.05), *p < 0.05, **p < 0.01, ***p < 0.001.

Studies have shown that miR-155-5p can regulate the polarization of macrophages (27). Therefore, we speculated that Me49-DC-Exo might play a role in macrophage polarization through controlling miR-155-5p. To test this hypothesis, RAW264.7 cells were directly transfected with miR-155-5p mimics/inhibitors, and the proportion of M1 and M2 macrophages was measured using flow cytometry. Compared to the control group, transfection of miR-155-5p mimics significantly increased the percentage of CD86^+^ CD206^−^ M1 macrophages and decreased the percentage of CD86 ^−^ CD206 ^+^ M2 macrophages (*p* < 0.01). By contrast, transfection with miR-155-5p inhibitor reduced the percentage of CD86 ^+^ CD206 ^−^ M1 macrophages (*p* < 0.05) (**Figure 6A**). qRT-PCR analysis showed that mRNA levels of INOS (*p* < 0.01), IRF5 (*p* < 0.01), and TNF-α (*p* < 0.001) were significantly increased, and those of Arg-1 (*p* < 0.05), TGM2 (*p* < 0.01), and IL - 10 (*p* < 0.05) were significantly decreased in RAW264.7 cells transfected with miR-155-5p mimics compared with the control group (**Figure 6B**). However, mRNA levels of INOS (*p* < 0.001) and TNF-α (*p* < 0.01) were significantly lowered, and mRNA levels of TGM2 (*p* < 0.05) and IL-10 (*p* < 0.01) significantly raised in RAW264.7 cells transfected with miR-155-5p inhibitors (**Figure 6B**). These data suggest that miR-155-5p can trigger macrophage polarization toward the M1 phenotype.

### SOCS1 Is a Direct Target of miR-155-5p

**Figure 7:**
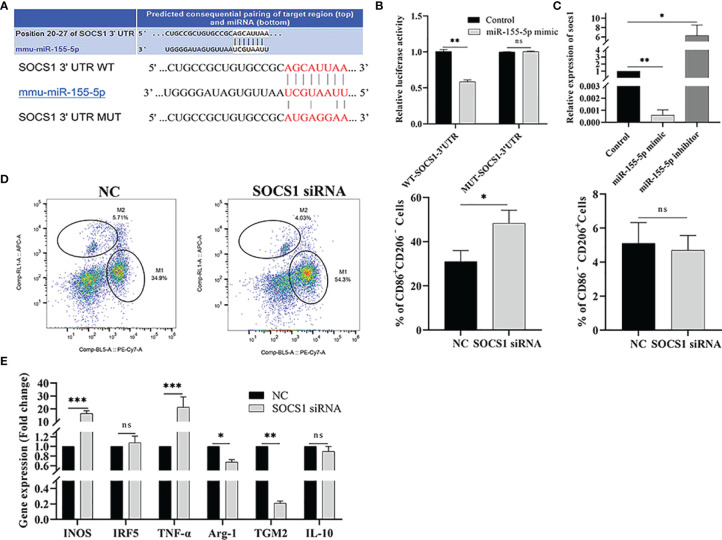
miR-155-5p regulates Raw264.7 cell polarization by targeting SOCS1. **(A)** Binding sequence between miR-155-5p and SOCS1 predicted by bioinformatics online analysis tool TargetScan. **(B)** The luciferase activity of HEK293 cells co-transfected with miR-155-5p mimics and luciferase reporter vectors containing wild type and mutant SOCS1 3 ‘UTR. **(C)** miR-155-5p mimics and inhibitors were transfected into Raw 264.7 cells, and SOCS1 expression in Raw264.7 cells was determined by qRT-PCR. **(D)** The proportion of CD86^+^ CD206 ^−^ M1 and CD86 ^−^ CD206 ^+^ M2 macrophages in Raw264.7 cells detected by flow cytometry after transfection with SOCS1-specific siRNA. **(E)** The mRNA levels of characteristic genes of M1 (INOS, TNF-α and IRF5) and M2 (TGM2, IL-10 and Arg-1) macrophages in Raw264.7 cells treated with SOCS1-siRNA were detected by qRT-PCR. ns (p ≥ 0.05), *p < 0.05, **p < 0.01, ***p < 0.001.

To explore the mechanism of miR-155-5p promoting polarization, the target genes of miR-155-5p were predicted by the Targetscan software. The results showed that the target gene of miR-155-5p was SOCS1, and there was a binding site between miR-155-5p and the 3’-UTR of SOCS1 (**Figure 7A**). We used a double luciferase reporter gene assay to verify the predicted results. Compared to the control group, miR-155-5p inhibited luciferase activity of WT-SOCS1-3’-UTR co-transfected with miR-155-5p (*p* < 0.01). However, luciferase activity of MUT-SOCS1-3’-UTR was not affected (**Figure 7B**), indicating that SOCS1 is the target gene of miR-155-5p. RAW264.7 cells were transfected with miR-155-5p, and SOCS1 mRNA level was detected by qRT-PCR. The mRNA level of SOCS1 was significantly reduced in RAW264.7 cells transfected with miR-155-5p mimics (*p* < 0.01). When RAW264.7 cells were transfected with miR-155-5p inhibitor, SOCS1 mRNA level was significantly increased (*p* < 0.05) (**Figure 7C**). These results suggest that SOCS1 is the target gene of miR-155-5p.

RNA interference technology was used to test whether miR-155-5p regulates macrophage polarization *via* target gene SOCS1. RAW264.7 cells were transfected with SOCS1-specific siRNA, and flow cytometry and qRT-PCR detected the M1/M2 macrophage-related markers and gene expression, respectively. The expression of CD86^+^ CD206^−^ was significantly increased in SOCS1-siRNA transfected cells (*p* < 0.05), and the expression level of CD86 ^−^ CD206 ^+^ in M2 macrophages was significantly lower than that in the control group (*p* < 0.01) (**Figure 7D**). The qRT-PCR results showed that the mRNA levels of INOS and TNF-*α*, (M1 macrophage-specific genes) were significantly up-regulated in RAW264.7 cells transfected with SOCS1-siRNA (*p* < 0.001) compared with the control group. The expression of M2 macrophage-specific genes, Arg-1 (*p* < 0.05) and TGM2 (*p* < 0.01), was significantly reduced (**Figure 7E**).

## Discussion

In this study, DC-Exo and Me49-DC-Exo were successfully isolated from cell supernatants. We found that Me49-DC-Exo lowered tumor growth by inhibiting M2 macrophage polarization in a tumor-bearing mouse model of colorectal cancer. When RAW264.7 cells were treated with membrane-dye-labeled exosomes, Me49-DC-Exo was shown to be internalized by macrophages, thus promoting macrophage M1 polarization while inhibiting macrophage M2 polarization. *T. gondii* infection significantly increased miR-155-5p expression in exosomes, which regulates macrophage polarization. When miR-155-5p mimics were transfected into RAW264.7 cells, the proportion of M1 macrophages increased, while the proportion of M2 macrophages decreased. However, when the expression of miR-155-5p was suppressed by miR-155-5p inhibitor transfection, the proportion of M2 macrophages in RAW264.7 cells was increased, while that of M1 macrophages was declined. miR-155-5p regulated macrophage polarization through SOCS1, a direct target of miR-155-5p as shown by bioinformatic analysis.

Targeting macrophage polarization is a successful anti-tumor strategy (28, 29). Rolny et al. (30) demonstrated that host-produced histidine-rich glycoprotein (HRG) reduced tumor growth and metastasis by modulating TAM polarization from M2 to M1 phenotype. Paclitaxel, an antineoplastic agent, is used to treat ovarian cancer by reprogramming TAM into M1 phenotype *via* TLR4-signalling (31). Lai et al. (32) showed that the long non-coding RNA NBR2 suppressed the progression of colorectal cancer *in vitro* and *in vivo* by regulating the polarization of TAM. In this study, Me49-DC-Exos co-cultured with RAW264.7 cells *in vitro* increased the fraction of CD86^+^ CD206 ^−^ cells and mRNA levels of INOS and TNF-α, suggesting that Me49-DC-Exo contributes to M1 polarization. Me49-DC-Exo suppressed M2 macrophage polarization by decreasing the proportion of CD86 ^−^ CD206 ^+^ cells and the mRNA levels of related genes Arg-1 and IL-10, which was consistent with the results of *in vivo* experiment. Our results from a tumor-bearing mouse model of colorectal cancer support the negative correlation between the inhibition of M2 macrophages by Me49-DC-Exo and tumor growth.

Exosomes, through delivering bioactive molecules to recipient cells, play a crucial regulatory role in cell signal transduction and biological functions (33). Exosomes derived from the plasma of *Plasmodium*-infected mice could bind to and lower the expression of vascular endothelial growth factor receptor 2 (VEGFR2) *via* miRNA 16-5p/17-5p/322-5p/497-5p, thereby inhibiting tumor growth by anti-angiogenesis (34). The miR-934 in exosomes derived from colorectal cancer cells triggered M2 macrophages polarization, promoting hepatic metastasis of colorectal cancer (35). These results imply that miRNAs in exosomes play an essential role in colorectal cancer disease progression. Here, high-throughput sequencing analysis revealed significant alteration in the abundance of miRNA 155-5p/155-3p/182-5p/125b-2-3p/9-5P in exosomes derived from DC infected with *T. gondii* Me49 strain. miR-155-5p, miR-155-3p, miR-125b-2-3p, and miR-182-5p were significantly overexpressed, but miR-9-5p was significantly down-regulated in the Me49-DC-Exo group compared with the DC-Exo group. Except for miRNA 125b-2-3p, the recorded differentially expressed miRNAs in exosomes in our study differed from those reported by Li et al. (36) in exosomes derived from DC2.4 dendritic cells infected with *T. gondii* RH strain tachyzoite. During the formation process, exosomes capture some components of the infected host cell, including *T. gondii* antigenic material. As a result, we hypothesize that the observed variation in miRNAs differential expression is due to the difference between RH and Me49 strains. When infecting DC, the exosomes generated by the two strains would vary. Another explanation might be the differences in the DC type. In this study bone marrow-derived DC were infected with *T. gondii*, whereas Li et al. (36) used DC2.4 dendritic cells, which are immortalized murine dendritic cells generated by transducing C57BL/6 mouse bone marrow isolates with retrovirus vectors expressing murine granulocyte-macrophage CSF as well as the oncogenes MYC and RAF (37).

SOCS1, a member of the suppressor of cytokine signaling family, is a key molecular switch mediating the signaling pathway of macrophage polarization. SOCS1 is up-regulated in M2 macrophages, and this up-regulation is critical for maintaining M2 phenotype and function (38). The regulation of M2 macrophage polarization by SOCS1 is mediated by signal transducer and activator of transcription 1 (STAT1) activation. Simultaneously, SOCS1 contains the SH2 (Src Homology 2) functional domain, which can inhibit STAT1 activity (39). The SOCS1/STAT1 pathway played a critical role in inhibiting the growth of prostate cancer by regulating macrophage polarization in the tumor microenvironment (40). Liang et al. (41) revealed the importance of the SOCS1/STAT1 pathway in macrophage polarization towards the M1 phenotype. Cai et al. (42) showed that SOCS1/STAT1 is one of the most crucial pathways for exosomal miR-221 to promote the polarization of M1 macrophages. In this study, miR-155-5p in exosomes derived from *T. gondii* Me49 strain-infected DC affected macrophage polarization by controlling the expression level of target gene SOCS1. However, further studies are required to determine whether miR-155-5p in exosomes regulates macrophage polarization through the SOCS1/STAT1 pathway.

In conclusion, our results demonstrated that Me49-DC-Exo effectively inhibited tumor growth in a tumor-bearing mouse model of colorectal cancer by regulating macrophage polarization. Through SOCS1, Me49-DC-Exo enriched with miR-155-5p could induce macrophage polarization to M1 phenotype. These findings establish the foundation for future research using miR-155-5p in Me49-DC-Exo for colorectal cancer treatment.

## Data Availability Statement

The datasets presented in this study can be found in online repositories. The name of the repository and accession number can be found below: NCBI (https://www.ncbi.nlm.nih.gov); GSE197114.

## Ethics Statement

The animal study was reviewed and approved by the Animal Care and Use Committee of Shanghai Veterinary Research Institute, the Chinese Academy of Agricultural Sciences

## Author Contributions

SZ executed experiments, interpreted results, and wrote the manuscript. ZL and JL designed and supervised the experiments. AA revised the manuscript. TZ and XC co-wrote the manuscript. HG, RM, and YH provided suggestion. GL, ZL, and ZC analyzed data, revised the manuscript and supervised the study. All authors read and approved the final manuscript.

## Funding

This study was supported in part by National Natural Science Foundation of China (Grant No. 31302083, 31672541), the Shanghai Science and Technology Commission Scientific Research Project (Grant No. 20140900400), Shanghai Agriculture Applied Technology Development Program, China (Grant No. 2019-02-08-00-08-F01151), National Key Research and Development Program of China (Grant No. 2017YFD0500401), and Shanghai Science and Technology Commission Popular Science Project (Grant No. 20DZ2304800).

## Conflict of Interest

The authors declare that the research was conducted in the absence of any commercial or financial relationships that could be construed as a potential conflict of interest.

## Publisher’s Note

All claims expressed in this article are solely those of the authors and do not necessarily represent those of their affiliated organizations, or those of the publisher, the editors and the reviewers. Any product that may be evaluated in this article, or claim that may be made by its manufacturer, is not guaranteed or endorsed by the publisher.
