# Corrigendum: Anti-Tumoral Effect and Action Mechanism of Exosomes Derived From *Toxoplasma gondii*-Infected Dendritic Cells in Mice Colorectal Cancer

**DOI:** 10.3389/fonc.2022.927917

**Published:** 2022-06-21

**Authors:** Shilan Zhu, Jinmiao Lu, Zhibing Lin, Asmaa M. I. Abuzeid, Xiaoyu Chen, Tingting Zhuang, Haiyan Gong, Rongsheng Mi, Yan Huang, Zhaoguo Chen, Guoqing Li

**Affiliations:** ^1^ Guangdong Provincial Key Laboratory of Zoonosis Prevention and Control, College of Veterinary Medicine, South China Agricultural University, Guangzhou, China; ^2^ Key Laboratory of Animal Parasitology of Ministry of Agriculture, Laboratory of Quality and Safety Risk Assessment for Animal Products on Biohazards (Shanghai) of Ministry of Agriculture, Shanghai Veterinary Research Institute, Chinese Academy of Agricultural Sciences, Shanghai, China; ^3^ School of Agriculture and Biology, Shanghai Jiao Tong University, Shanghai, China; ^4^ Faculty of Veterinary Medicine, Suez Canal University, Ismailia, Egypt

**Keywords:** *Toxoplasma gondii*, dendritic cells, exosome, miRNA, macrophage, miR-155-5p

In the original article, there was a mistake in **Figure 2** as published. 1. **Figure 2A** (left) missed to indicate the corresponding groups for the panels. The corresponding groups are now correctly indicated. 2. **Figure 2B**: The representative flow cytometry plot was repeated two times (same flow cytometry image shown in the third column). The flow cytometry image are the results of flow cytometry detection of anti-CD86-PC7 in the Me49 group.We replaced the lower flow cytometry image in the third column with a representative flow-cytometry plot of anti-CD206-APC in the Me49 group**.** The corrected **Figure 2** appears below.

**Figure 2:**
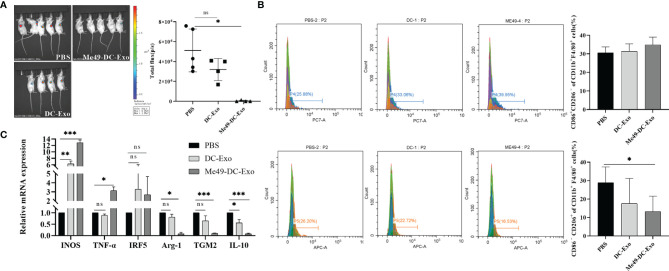
Me49-DC-Exo inhibited tumor growth in mouse and regulated macrophage polarization. **(A)** On day 4 after treatment, the IVIS imager detected bioluminescence images in tumors of mouse and quantified the bioluminescence signal intensity of each tumor in mouse. **(B)** Flow cytometry was used to label CD86 + or CD206 +, and CD45 + CD11b + F4/80 + macrophages in blood of mice injected with DC-Exo and Me49-DC-Exo were stained to detect the percentage of CD86+ CD206 − M1 macrophages and CD86 − CD206 + M2 macrophages. **(C)** mRNA levels of M1 macrophage specific genes (INOS, TNF-a, and IRF5) and M2 macrophage specific genes (TGM2, Arg-1 and IL10) in blood of tumor-bearing mice injected intratumorally with PBS, DC-Exo and Me49-DC-Exo were detected by qRT-PCR. The data were expressed as mean ± standard deviation, and the independent sample t-test was used to compare the statistical differences between two groups. ns (p ≥ 0.05), *p < 0.05, **p < 0.01, ***p < 0.001.

The authors apologize for this error and state that this does not change the scientific conclusions of the article in any way. The original article has been updated.

## Publisher’s Note

All claims expressed in this article are solely those of the authors and do not necessarily represent those of their affiliated organizations, or those of the publisher, the editors and the reviewers. Any product that may be evaluated in this article, or claim that may be made by its manufacturer, is not guaranteed or endorsed by the publisher.

