# Supplementary material for: Anti-Tumoral Effect and Action Mechanism of Exosomes Derived From Toxoplasma gondii-Infected Dendritic Cells in Mice Colorectal Cancer
Source: Front Oncol. 2022 Apr 22;12:870528. doi: 10.3389/fonc.2022.870528 (PMC9118538; doi:10.3389/fonc.2022.870528)
Supplement: Supplementary file 1 [file Table_1.docx]

**Additional file**

**Table S1** Primers used for the quantitative real-time PCR in this study

| Target gene | Primer name | Primer sequence  (5’- 3’) | Fragment size | References |
| --- | --- | --- | --- | --- |
| GAPDH （60℃） | G-F  G-R | TGGATTTGGACGCATTGGTC  TTTGCACTGGTACGTGTTGAT | 211 | Kamat et al.（2015） |
| INOS （60℃） | N-F  N-R | TGGTGAAGGGACTGAGCTGT  CGTTCTCCGTTCTCTTGCAG | 254 | Adah et al.（2019） |
| IRF5  （60℃） | I-F  I-R | CCTCAGCCGTACAAGATCTACGA  GTAGCATTCTCTGGAGCTCTTCCT | 96 | Al Mamun et al. （2019） |
| IL-10  （60℃） | 10-F  10-R | GTAGAAGTGATGCCCCAGGC  GGGGAGAAATCGATGACAGC | 101 | Adah et al.（2019） |
| TGM2（60℃） | T-F  T-R | GACAATGTGGAGGAGGGATCT  CTCTAGGCTGAGACGGTACAG | 120 | Annas et al.（2021） |
| Arg-1（60℃） | A-F  A-R | CTGAGAGATTCAAGGCAAGAGG  GAACGCGCTATCTTACCCCAG | 173 | Wang et al.（2012） |
| TNF-α  (60℃) | α-F  α-R | CCGATTTGCCACTTCATACCA  TAGGGCAAGGGCTCTTGATG | 116 |  |
| SOCS1（60℃） | S1-F  S1-R | CTGCGGCTTCTATTGGGGAC  AAAAGGCAGTCGAAGGTCTCG | 216 | Yang et al*.*（2020） |
